# Serum regucalcin is a useful indicator of liver injury severity in patients with hepatitis B virus-related liver diseases

**DOI:** 10.1590/1414-431X20198845

**Published:** 2019-09-30

**Authors:** Xinhuan Wei, Haibin Yu, Peng Zhao, Li Xie, Li Li, Jing Zhang

**Affiliations:** 1 Department of Hepatitis C and Drug-Induced Liver Disease, Beijing Youan Hospital, Capital Medical University, Beijing, China; 2 Center of Minimally Invasive Interventional Therapy, Beijing Youan Hospital, Capital Medical University, Beijing, China; 3 Beijing Institute of Hepatology, Capital Medical University, Beijing, China; 4 Center for Infectious Diseases, Beijing Youan Hospital, Beijing Key Laboratory for HIV/AIDS Research, Beijing, China

**Keywords:** Regucalcin, Liver injury, Chronic hepatitis B infection, ELISA, Liver failure

## Abstract

Regucalcin is a soluble protein that is principally expressed in hepatocytes. Studies of regucalcin have mainly been conducted in animals due to a lack of commercially available kits. We aimed to develop an enzyme-linked immunosorbent assay (ELISA) to quantify serum regucalcin in patients with hepatitis B virus (HBV)-related disease. High-titer monoclonal antibodies and a polyclonal antibody to regucalcin were produced, a double-antibody sandwich ELISA method was established, and serum regucalcin was determined in 47 chronic hepatitis B (CHB) patients, 91 HBV-related acute-on-chronic liver failure (HBV-ACLF) patients, and 33 healthy controls. The ELISA demonstrated an appropriate linear range, and high levels of reproducibility, sensitivity, specificity, accuracy, and stability. The median serum regucalcin concentrations in HBV-ACLF and CHB patients were 5.46 and 3.76 ng/mL, respectively (P<0.01), which were much higher than in healthy controls (1.72 ng/mL, both P<0.01). For the differentiation of CHB patients and healthy controls, the area under curve (AUC) was 0.86 with a cut-off of 2.42 ng/mL, 85.7% sensitivity, and 78.8% specificity. In contrast, the AUC of alanine aminotransferase (ALT) was lower (AUC=0.80, P=0.01). To differentiate ACLF from CHB, the AUC was 0.72 with a cut-off of 4.26 ng/mL, 77.0% sensitivity, and 61.2% specificity while the AUC of ALT was 0.41 (P=0.07). Thus, we have developed an ELISA that is suitable for measuring serum regucalcin and have shown that serum regucalcin increased with the severity of liver injury due to HBV-related diseases, such that it appears to be more useful than ALT as a marker of liver injury.

## Introduction

Evaluation of the severity of liver injury is important in the diagnosis and treatment of liver diseases. Serum alanine aminotransferase (ALT) concentration is the most widely-used index of liver injury, but it does not reflect the severity of liver pathology in some patients (1). Although the severity of liver injury can be judged using several indices, such as bilirubin and the international normalized ratio (INR), new serum markers of liver injury are urgently required.

Regucalcin, which is also referred to as senescence marker protein-30 (SMP30), is principally expressed in hepatocytes (2). Regucalcin has a number of roles in cells, such as in the maintenance of intracellular calcium homeostasis (3), suppression of calcium-dependent signaling proteases (4), participation in the biosynthesis of vitamin C (5), and suppression of apoptosis (6). A few previous studies have shown that serum regucalcin concentrations increase both in animal models of liver injury (7) and patients with chronic hepatitis (8), but these studies have been hampered by the lack of availability of commercial kits for the measurement of regucalcin.

In the present study, we aimed to establish a double-antibody sandwich enzyme-linked immunosorbent assay (ELISA) to measure regucalcin and to evaluate the usefulness of serum regucalcin for the evaluation of liver injury, by measuring the serum regucalcin concentrations in patients with hepatitis B virus (HBV)-induced diseases [chronic hepatitis B (CHB) and HBV-related acute-on-chronic liver failure (HBV-ACLF)].

## Material and Methods

### Preparation and purification of regucalcin protein and antibodies

The regucalcin antigenic epitope was analyzed using DNA Star software (DNA Star, Inc., USA), and then the corresponding fragment of the gene (amino acids: Q84–G299) was amplified by PCR using the forward primer 5′-CCGGAATTCCAATCAGCAGTTGTCTTGGCCAC-3′ and reverse primer 5′-ATTTGCGGCCGCTTATCATCCCGCATAGGAG-3′. The fragment was inserted into the prokaryotic expression vector pGEX-4T-1 (Pharmacia, USA). The regucalcin gene was then expressed in *E. coli* (Beijing Dingguo Changsheng Biotechnology Co. Ltd., China) and the regucalcin recombinant protein was purified by ion-exchange chromatography (GE Healthcare, USA). New Zealand white rabbits (Laboratory Animal Center of the Academy of Military Medical Sciences, China) were injected several times at different sites with the recombinant protein, emulsified with Freund's adjuvant (Sigma-Aldrich LLC., USA). Blood samples were collected on days 7–10 after the final injection, centrifuged at 5000 *g* for 10 min at 4°C, and the polyclonal antibody (pAb) against regucalcin was purified by precipitation.

Monoclonal antibodies (mAbs) were produced using two hybridoma cell lines. The cells were injected into the peritoneal cavity of liquid paraffin-treated BALB/c mice (Laboratory Animal Center of the Academy of Military Medical Sciences) to generate mAbs. Ascitic fluid was collected on days 10–14 after the injection and the mAbs were purified by precipitation (mAb A and mAb B).

### Establishment of a regucalcin double-antibody sandwich ELISA

To develop a highly sensitive sandwich ELISA, we used chessboard titration to determine the appropriate antibodies to use for capture and detection out of the two mAbs and pAb generated. After optimizing the primary antibodies and secondary goat-anti-rabbit/mouse antibodies, which were conjugated to horseradish peroxidase (HRP), we created a double-antibody sandwich ELISA, in which we used mAb A as the capture antibody and the pAb as the detection antibody. After trying several combinations, we established the optimal experimental conditions. The plate was coated with 100 μL of mAb A diluted in 0.5 μg/mL and left overnight at 4°C. After blocking and washing, it was incubated with commercial regucalcin protein standard dilutions (Abcam, USA, 2.3–75 ng/mL, 100 μL) or serum (100 μL) overnight at 4°C, which was followed by the addition of pAb (1 μg/mL, 100 μL) and goat-anti-rabbit antibody conjugated to HRP (diluted 1:10,000, 100 μL). The 96-well microplate was agitated for 90 min at 37°C and then read at 450/630 nm within 30 min. The concentration of serum regucalcin was then calculated using a linear standard curve.

### Evaluation of the double-antibody sandwich ELISA

We evaluated the double-antibody sandwich ELISA using the guidelines for ELISA kits (YY/T 1183-2010) (9) issued by the China Food and Drug Administration (cFDA): 1) *Linearity*: The commercial regucalcin protein was tested at a number of concentrations (150, 75, 37.50, 18.75, 9.37, 4.69, 2.34, and 1.17 ng/mL) to determine the range of linearity. 2) *Reproducibility*: Commercial regucalcin (3 ng/mL) was assayed 10 times and the coefficient of variation (CV) was determined by dividing the standard deviation (SD) by the mean and multiplying by 100, giving an indication of the repeatability of the method. 3) *Sensitivity*: Sample diluents (that is 0 ng/mL) were assayed 20 times and the limit of detection (LoD) was calculated using the mean and SD of the blank absorbance (Ab) value as LoD=average absorbance of the blank + 3×SD (10). 4) *Specificity*: Because glutathione S transferase (GST) protein was present in the synthesized recombinant regucalcin protein preparation, it was also used at various concentrations to test the specificity of the kit. 5) *Stability*: The coated ELISA kits were stored at 37°C or 4°C for 1 week, after which their detection efficacies were compared.

### Patient recruitment

Forty-seven CHB patients and 91 HBV-ACLF patients were consecutively and prospectively recruited between June 2018 and March 2019. CHB was diagnosed according to the published guidelines for hepatitis B (11) and ACLF was diagnosed with reference to the Asian Pacific Association for the Study of the Liver (12) definition. Briefly, the criteria were: chronic liver disease, serum bilirubin >10× the upper limit of normal (ULN), INR >1.5 or prothrombin activity (PTA) ≤40%, ascites, or encephalopathy for 4 weeks. Patients with hepatocellular carcinoma, other causes of liver injury, such as alcoholic liver diseases, autoimmune liver diseases, non-alcoholic fatty liver diseases, or HIV/HBV or HCV/HBV co-infection were excluded. Thirty-three healthy, age- and sex-matched individuals were recruited as controls. All of the participants provided written informed consent. The protocol was approved by the Ethics Committee of Beijing Youan Hospital, Capital Medical University (NO [2018]019) and conformed to the guidelines of the Helsinki Declaration. The fasting blood samples were centrifuged at 1250 *g* for 10 min at 25°C, and serum was collected and stored at −80°C.

### Statistical analysis

Statistical analysis was performed using SPSS 16.0 (SPSS Inc., USA). All results are reported as medians (25 to 75th percentiles). Comparisons among groups were made using the Kruskal-Wallis or Mann-Whitney tests. Spearman's correlations were used to evaluate linear relationships between continuous variables. The diagnostic performance of an assay was assessed by analysis of the receiver-operating characteristic (ROC) curve. The assessment of sensitivity and specificity was made using selected cut-off values. P<0.05 was considered to represent statistical significance.

## Results

### Evaluation of the double-antibody sandwich ELISA

**Figure 1:**
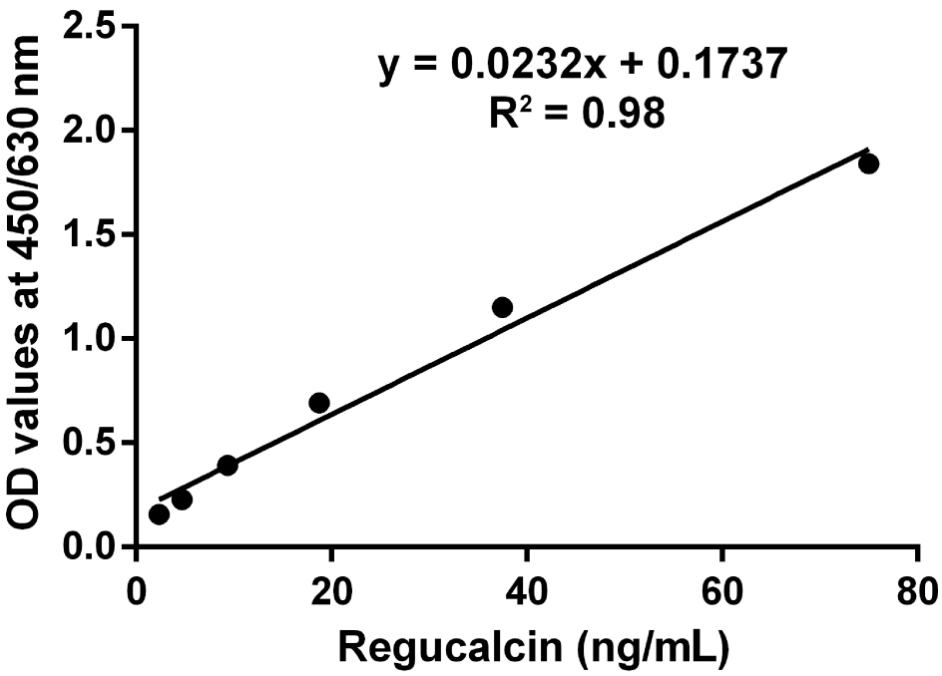
Standard curve for regucalcin constructed using absorbance (optical density, OD) values and serum regucalcin concentrations.

The concentrations of regucalcin protein, mAb A, mAb B, and pAb used were 323.4, 2.2, 2.3, and 2.0 mg/mL, respectively, and all were of >90% purity. Serum regucalcin was measured in the range 1.17–150 ng/mL. The equation for the standard curve constructed was y = 0.0232x + 0.1737 and the absorbances and concentrations of the regucalcin standards were correlated with r^2^=0.98 (Figure 1). The LoD was 0.76 ng/mL and the CV was 7%, which is within the acceptable range (<10%) (13). Differences in Ab values were <14.7% when various concentrations of GST protein were tested. After storage at 37°C for 1 week, the Ab values were 2% higher than those obtained using a kit stored at 4°C, which met the required standard of <20%. Thus, all the relevant indices (linearity, sensitivity, specificity, reproducibility, and stability) achieved the most up-to-date standards for ELISA kits issued by the cFDA, and the method was awarded a Certificate of Invention Patent by the State Intellectual Property Office of the People's Republic of China (Patent number: ZL 2013 1 0206809.5).

### Relationship between serum regucalcin and other serum liver injury markers

**Figure 2:**
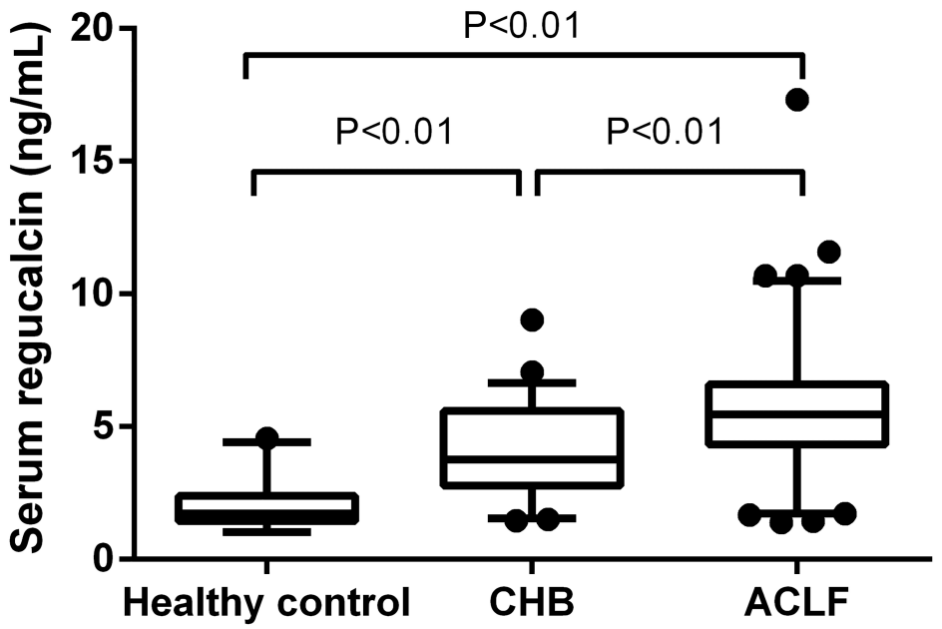
Serum regucalcin concentrations in heathy controls, and chronic hepatitis B (CHB) and acute-on-chronic liver failure (ACLF) patients. The top and bottom of each box represent the 25th and 75th percentiles. The line through the box is the median and the error bars represent the 5th and 95th percentiles. Statistical analysis was done with the Mann-Whitney test.

The clinical characteristics and laboratory data for the control participants, CHB patients, and ACLF patients are shown in Table 1. The serum concentrations of regucalcin in the three groups were 1.72 ng/mL (1.44–2.39 ng/mL), 3.76 ng/mL (2.78–5.59 ng/mL), and 5.46 ng/mL (4.32–6.58 ng/mL), respectively. HBV-ACLF patients had higher regucalcin concentrations than CHB patients (P*<*0.01) and the serum regucalcin concentrations in both HBV-ACLF and CHB patients were much higher than those in healthy controls (P<0.01 for both comparisons, Figure 2). In CHB and ACLF patients, serum regucalcin concentrations correlated weakly with aspartate aminotransferase (AST) (r=0.16, P=0.02), bilirubin (r=0.28, P<0.01), albumin (r=−0.23, P<0.01), cholinesterase (r=0.17, P=0.02), and PTA (r=−0.27, P<0.01).

**Table 1:** Clinical characteristics of the healthy controls and the chronic hepatitis B (CHB) and acute-on-chronic liver failure (ACLF) patients.

	Healthy controls	CHB patients	ACLF patients
N	33	47	91
Age (years)	38 (20–58)	35 (18–73)	45.5 (16–77)
Gender (male:female)	13:20	35:12	68:23
ALT (U/L)	18 (8–40)	53.3 (8.7–1095.3)	73.6 (12–1188)
AST (U/L)	21.0 (18.0–28.0)	49.8 (16.6–432.9)	90.65 (8–1192)
TBIL (μmol/L)	11.1 (4.0–16.0)	19.1 (8.0–753.9)	355.25 (13–965)
ALB (g/L)	42.1 (40.5–45.3)	38.2 (34.2–41.4)	32.8 (19–47)
PALB (mg/L)	–	117.4 (66.2–168.0)	65.7 (26–212)
CHE (U/L)	–	5845.0 (1539–13100)	3996.5 (975–8930)
TBA (μmol/L)	–	15.5 (1–276.3)	161.7 (93.6–229.2)
CREA (μmol/L)	62.0 (47.0–93.0)	68.1 (1.32–95.7)	65.1 (23–263)
BUN (mmol/L)	4.84 (3.26–46.80)	3.94 (2.03–40.40)	5.11 (1–39)
PTA (%)	95.5 (94–99.8)	91.4 (9.27–116.2)	35.3 (16.5–38.6)
INR	–	0.99 (0.84–1.34)	1.60 (1–4)
MELD	–	4.98 (–5.49–35.30)	20.5 (14.8–64.8)
Regucalcin (ng/mL)	1.72 (1.44–2.39)	3.76 (2.78–5.59)	5.46 (4.32–6.58)

ALT: alanine aminotransferase; AST: aspartate aminotransferase; TBIL: total bilirubin; ALB: albumin; PALB: pre-albumin; CHE: cholinesterase; TBA: total bile acid; CREA: creatinine; BUN: blood urea nitrogen; PTA: prothrombin activity; INR: international normalized ratio; MELD: model for end-stage liver disease. Data are reported as medians (25–75th percentiles).

### Clinical significance of regucalcin concentrations in CHB and ACLF patients

**Figure 3:**
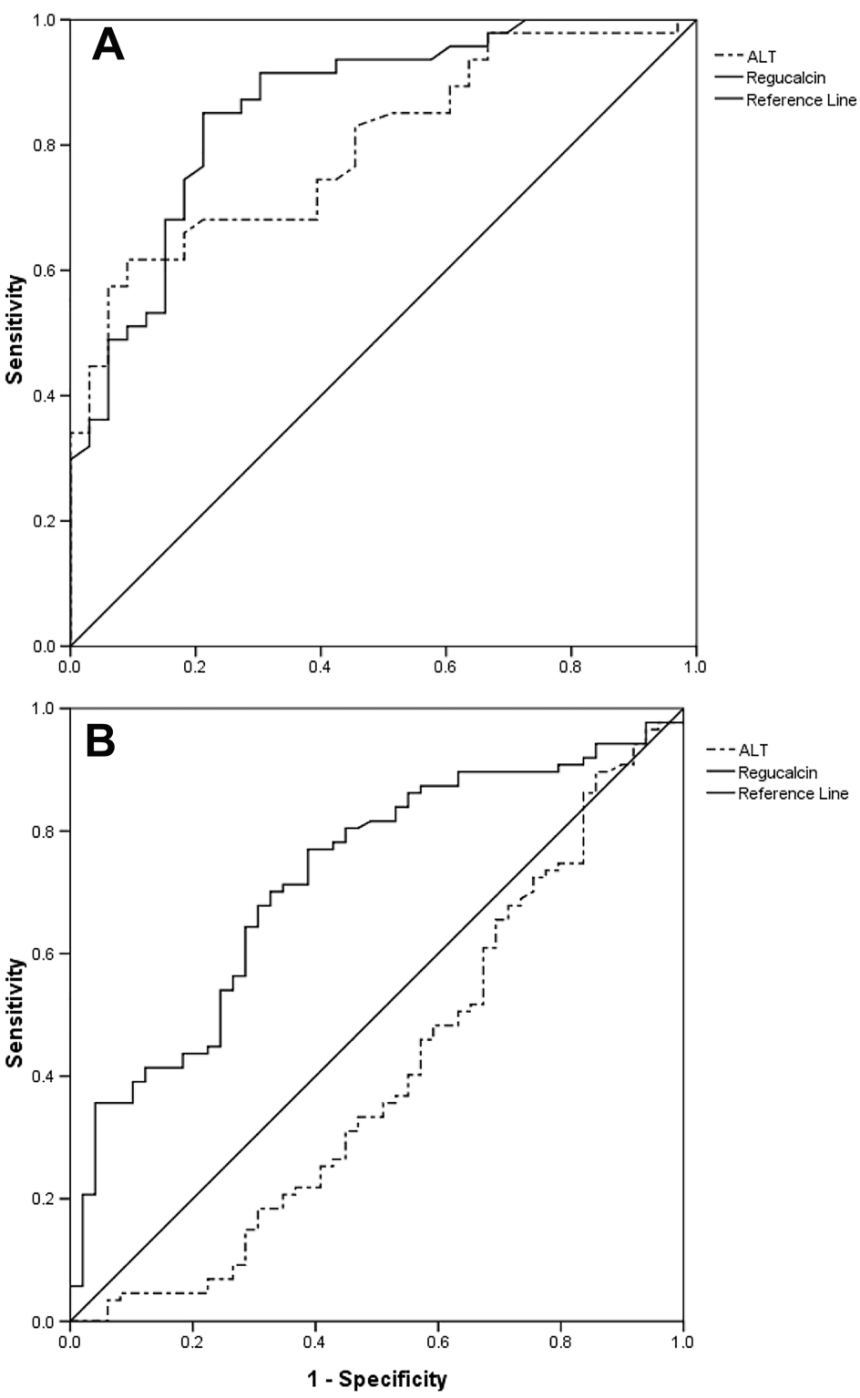
Receiver operating characteristic (ROC) curves for regucalcin (RGN) and alanine aminotransferase (ALT) in chronic hepatitis B (CHB) (**A**) and acute-on-chronic liver failure (ACLF) (**B**) patients. **A**, The area under the curve (AUC) for RGN was 0.86 with a cut-off of 2.42 ng/mL. The sensitivity and specificity were 85.7 and 78.8%, respectively. The AUC for ALT was 0.80 (95%CI: 0.70–0.89, P<0.01) with a cut-off of 28.30 U/L (sensitivity: 0.62 and specificity: 0.91), which is lower than the AUC for RGN (P=0.01). **B**, The AUC for RGN was 0.72 with a cut-off of 4.26 ng/mL. The sensitivity and specificity were 77.0 and 61.2%, respectively. In contrast, the AUC of ALT was only 0.41 (95%CI: 0.30–0.51, P=0.07) (P<0.01).

For the differentiation of patients with chronic hepatitis and healthy controls, the AUC of regucalcin was 0.86 (95% confidence interval (CI): 0.79–0.95, P*<*0.01) with a cut-off of 2.42 ng/mL, and the sensitivity and specificity were 85.7 and 78.8%, respectively. In contrast, the AUC of ALT was 0.80 (95%CI: 0.70–0.89, P<0.01) with a cut-off of 28.30 U/L (sensitivity: 0.62 and specificity: 0.91), which is lower than the AUC of regucalcin (P=0.01, Figure 3A). To differentiate ACLF from CHB patients, the AUC of regucalcin was 0.72 (95%CI: 0.63–0.81, P<0.01) with a cut-off of 4.26 ng/mL, and the sensitivity and specificity were 77.0 and 61.2% respectively, while the AUC of ALT was only 0.41 (95%CI: 0.30–0.51, P=0.07) (P<0.01, Figure 3B).

## Discussion

To our knowledge, this is the first study to measure serum regucalcin concentration in ACLF patients. We found that the concentrations of regucalcin were significantly higher in CHB patients than in healthy controls, and higher still in patients with HBV-ACLF, indicating that serum regucalcin concentration is related to the severity of liver injury.

Evaluation of the severity of liver injury is an important part of the assessment of liver disease, but no ideal serum marker of liver injury exists. ALT is the most frequently used marker of liver injury, but it does not always reflect ongoing inflammation in CHB patients (14). The study by Lai et al. (15) has shown that even in patients with persistently normal serum ALT, 34% have grade 2 or 3 liver inflammation. Furthermore, in CHB patients with ALT values less than two times the upper limit of normal, 49.2% have significant liver inflammation (grade ≥2) (16). In addition, in liver failure patients, serum ALT may even decrease.

A number of new markers of liver injury have been proposed, including microRNAs (17), cytokeratin-18 (CK18) (18,19), high mobility group box protein 1 (HMGB-1) (20,21) for viral hepatitis, drug induced liver disease (DILI), non-alcoholic steatohepatitis (NASH), and liver failure. However, none of these has been accepted universally.

Regucalcin was originally identified in 1978 by Yamaguchi et al. (22), and was shown to be the same protein as SMP30 in 1992 (23). The regucalcin gene is located on the p11.3 to q11.2 segment of the X chromosome (24), and it encodes a 34-kDa protein composed of 299 amino acids, which is expressed mainly in hepatocytes and renal tubular epithelia. It has been shown to protect liver cells from UV irradiation-induced apoptosis (25).

Several studies have evaluated regucalcin as a potential biomarker of liver injury. In a carbon tetrachloride (CCl_4_) liver injury model (7) , rats given CCl_4_ five times at 3-day intervals showed significant increases in serum regucalcin concentration between day 3 and day 30, while serum AST, ALT, and γ-glutamyl transferase concentrations increased at the same time, but returned to normal by day 18. Therefore, it was concluded that serum regucalcin reflected liver injury, and may be more suitable than ALT for the evaluation of ongoing damage. Isogai and colleagues also demonstrated a significant increase in serum regucalcin in rats treated with galactosamine (26). Finally, administration of CCl_4_ significantly reduces liver regucalcin and increases serum regucalcin concentration 24 h after administration, indicating that it is released into the serum from hepatocytes when the liver is injured (27).

To date, only two studies have measured serum regucalcin in patients with liver injury. In the first, the serum concentration was 3.7–69.6 ng/mL in patients with liver disorders, and it was undetectable in healthy controls (28), but it was detectable in 18 patients with normal ALT activities. In the other study, serum regucalcin concentrations in acute liver failure patients were 3.65±0.34 times higher than in healthy volunteers, as determined by western blotting (8).

Most previous studies have used western blotting to measure regucalcin concentrations, which is a semi-quantitative method. In the study conducted by Yamaguchi, the pAb used in the ELISA was prepared in a rabbit immunized with regucalcin protein (29), which may have influenced its specificity. In this study, we have successfully established a double-antibody sandwich ELISA method, using an mAb as the capture antibody and a pAb as the detection antibody, which is known to be the type of assay with the highest sensitivity and specificity (30). Compared with a pAb, an mAb can capture the specific target antigen with high affinity from a mixture of diverse proteins. Because the synthesized recombinant regucalcin protein contains GST protein, we used commercial regucalcin protein as the standard, which guarantees that regucalcin can be detected without interference from GST. The linearity, sensitivity, reproducibility, and stability of the assay all met the specified guidelines, guaranteeing the reliability of the measurement.

We have shown low concentrations of serum regucalcin in healthy controls, whereas regucalcin was undetectable in the study by Yamaguchi and coworkers (28). In patients with HBV-related disease, serum regucalcin concentration weakly correlated with biochemical markers of liver injury, such as AST and bilirubin, and with markers of liver function, such as albumin and cholinesterase, which further supports a relationship between serum regucalcin and liver injury. In addition, we have shown differing serum regucalcin concentrations between healthy controls and patients with chronic hepatitis or ACLF, indicating that serum regucalcin concentration is related to the severity of liver injury, and may represent a superior biomarker for liver injury to ALT. Specifically, a serum regucalcin concentration higher than 2.42 ng/mL indicated the possibility of liver injury, and a concentration higher than 4.26 ng/mL implied liver failure.

The principal limitation of this study was that liver biopsies were not performed. Therefore, the relationship between serum regucalcin concentration and liver pathology should be assessed in a future study.

In conclusion, we have produced a double-antibody sandwich ELISA that permitted the ready quantification of serum regucalcin. Serum regucalcin increased with the severity of liver injury, and was superior to ALT as a biomarker of active liver injury.
